# Supplementary material for: Global early replication disrupts gene expression and chromatin conformation in a single cell cycle
Source: Genome Biol. 2022 Oct 17;23:217. doi: 10.1186/s13059-022-02788-7 (PMC9575230; doi:10.1186/s13059-022-02788-7)
Supplement: Supplementary file 1 — Additional file 1. Contains Figures S1-5, which are supplementary data in support of the main figures. [file 13059_2022_2788_MOESM1_ESM.pdf]

Fig S1

A

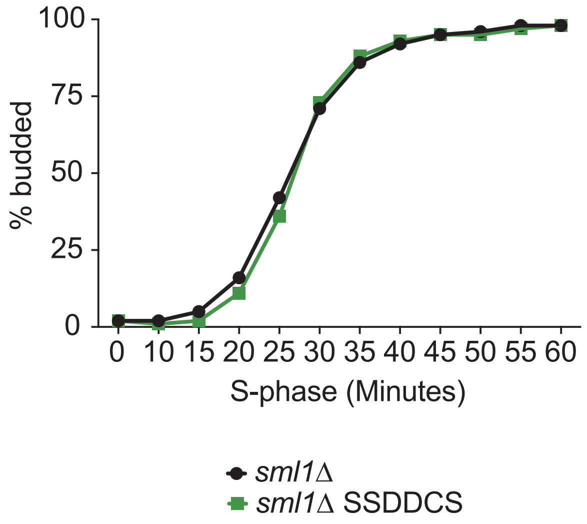
% budded
100
75
50
25
0
0 10 15 20 25 30 35 40 45 50 55 60
S-phase (Minutes)
sml1Δ
sml1Δ SSDDCS

B

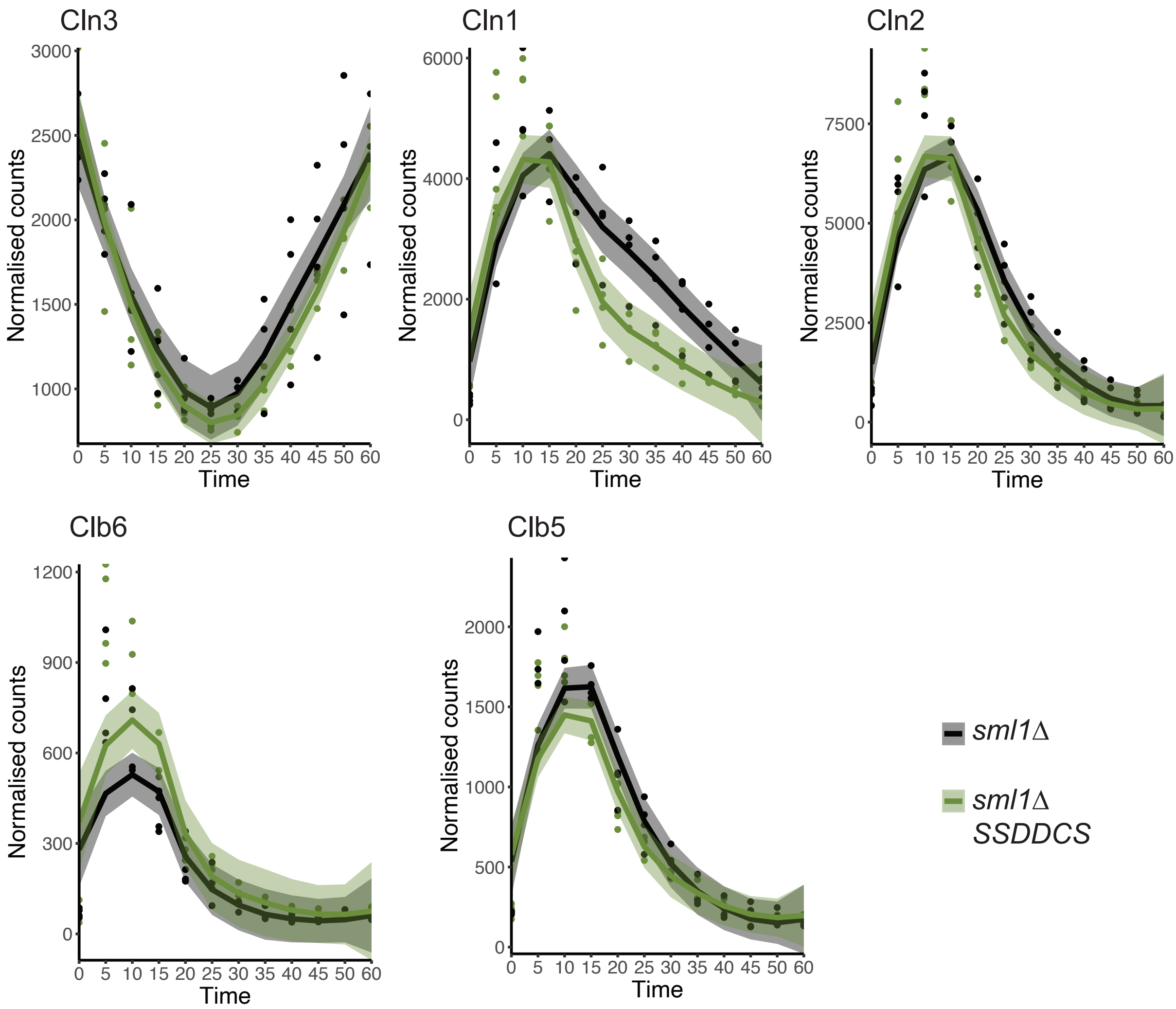
Cln3
Cln1
Cln2
Clb6
Clb5
Normalised counts
Time
0 5 10 15 20 25 30 35 40 45 50 60
sml1Δ
sml1Δ SSDDCS

Fig. S2

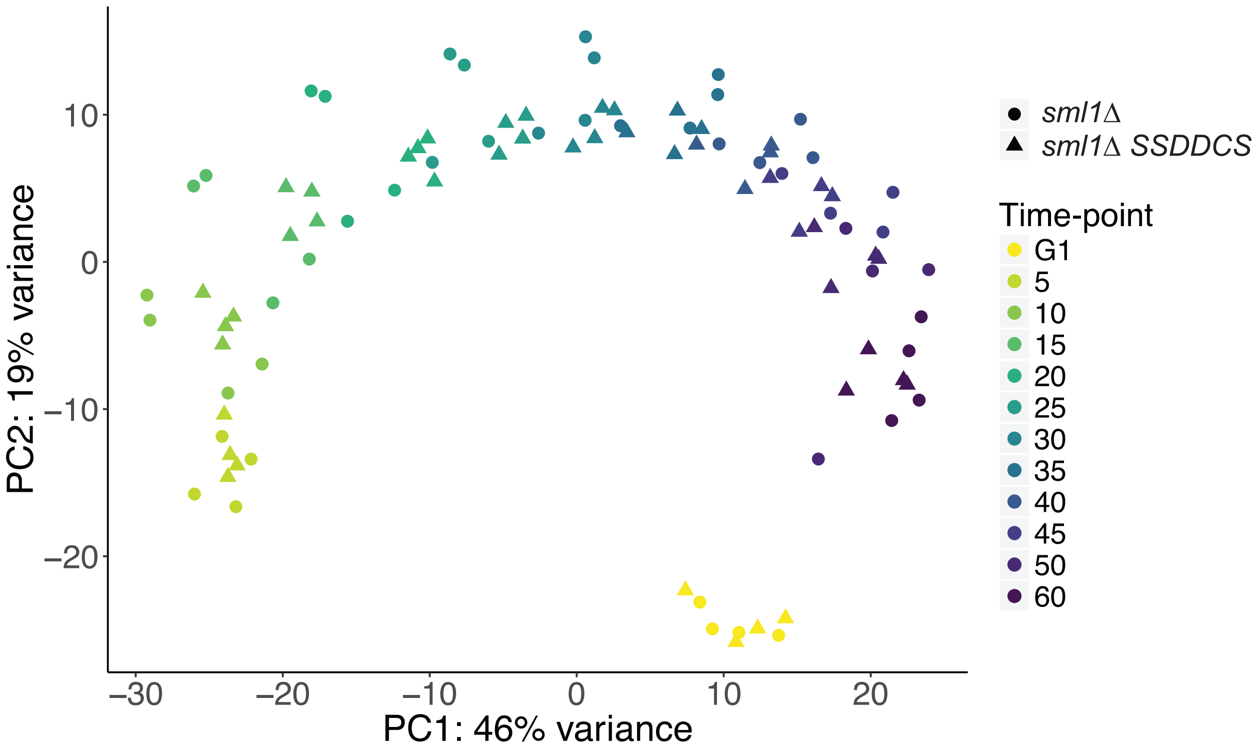
PC2: 19% variance
10
0
−10
−20
−30
−20
−10
0
10
20
PC1: 46% variance
sml1Δ
sml1Δ SSDDCS
Time-point
G1
5
10
15
20
25
30
35
40
45
50
60

Fig. S3

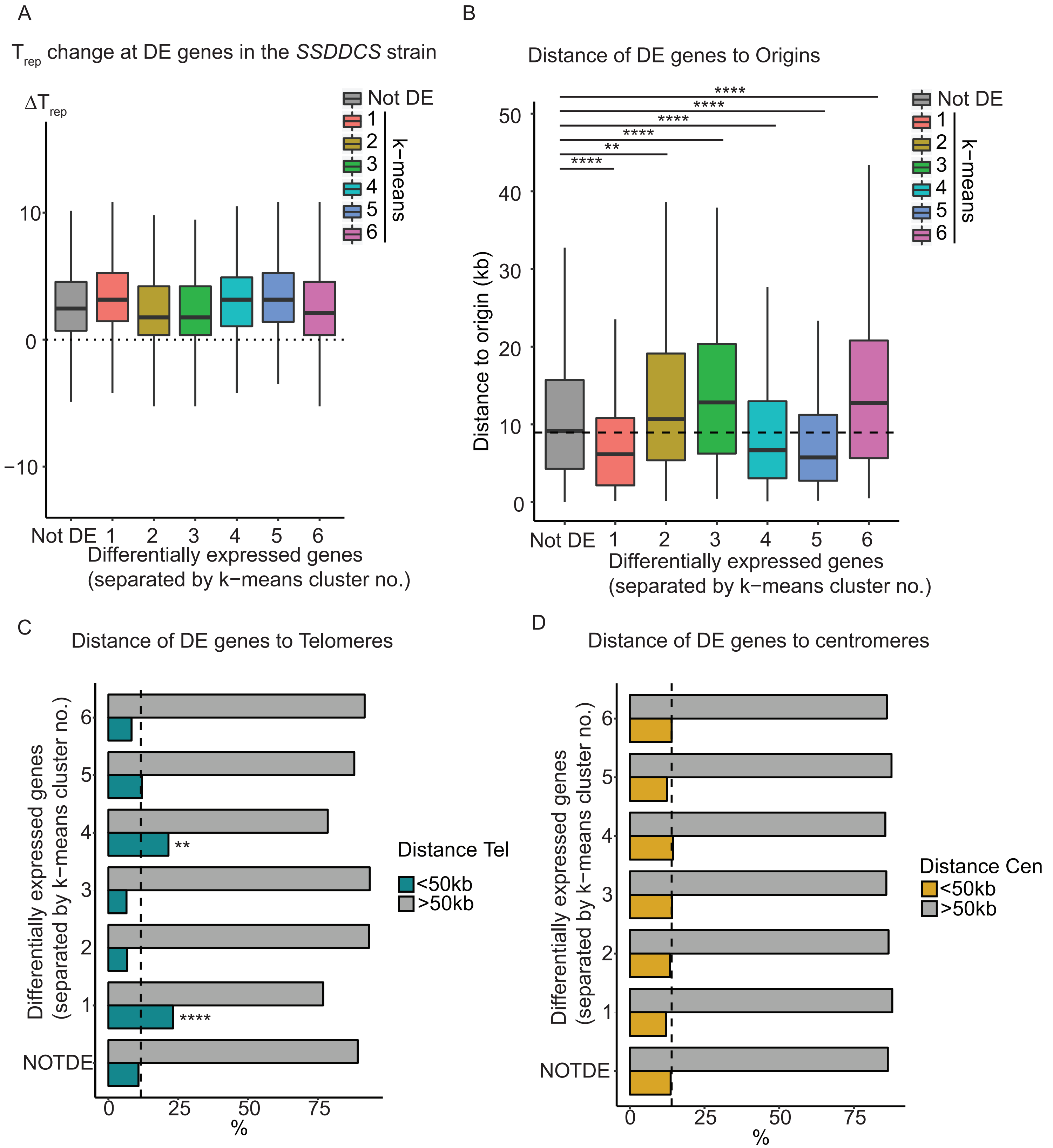
A
T_{rep} change at DE genes in the *SSDDCS* strain
ΔT_{rep}
Not DE
1
2
3
4
5
6
k−means
10
0
−10
Not DE 1 2 3 4 5 6
Differentially expressed genes
(separated by k−means cluster no.)
B
Distance of DE genes to Origins
****
****
****
****
**
****
Not DE
1
2
3
4
5
6
k−means
Distance to origin (kb)
50
40
30
20
10
0
Not DE 1 2 3 4 5 6
Differentially expressed genes
(separated by k−means cluster no.)
C
Distance of DE genes to Telomeres
Differentially expressed genes
(separated by k−means cluster no.)
6
5
4
3
2
1
NOTDE
**
****
0 25 50 75
%
Distance Tel
<50kb
>50kb
D
Distance of DE genes to centromeres
Differentially expressed genes
(separated by k−means cluster no.)
6
5
4
3
2
1
NOTDE
0 25 50 75
%
Distance Cen
<50kb
>50kb

Fig. S4

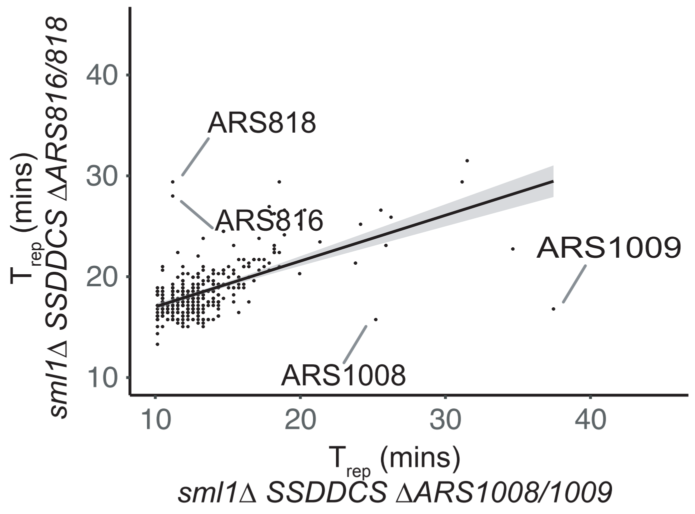
40
30
20
10
10
20
30
40
ARS818
ARS816
ARS1009
ARS1008
T_rep (mins)
sml1Δ SSDDCS ΔARS816/818
T_rep (mins)
sml1Δ SSDDCS ΔARS1008/1009

Supplementary Figure 5

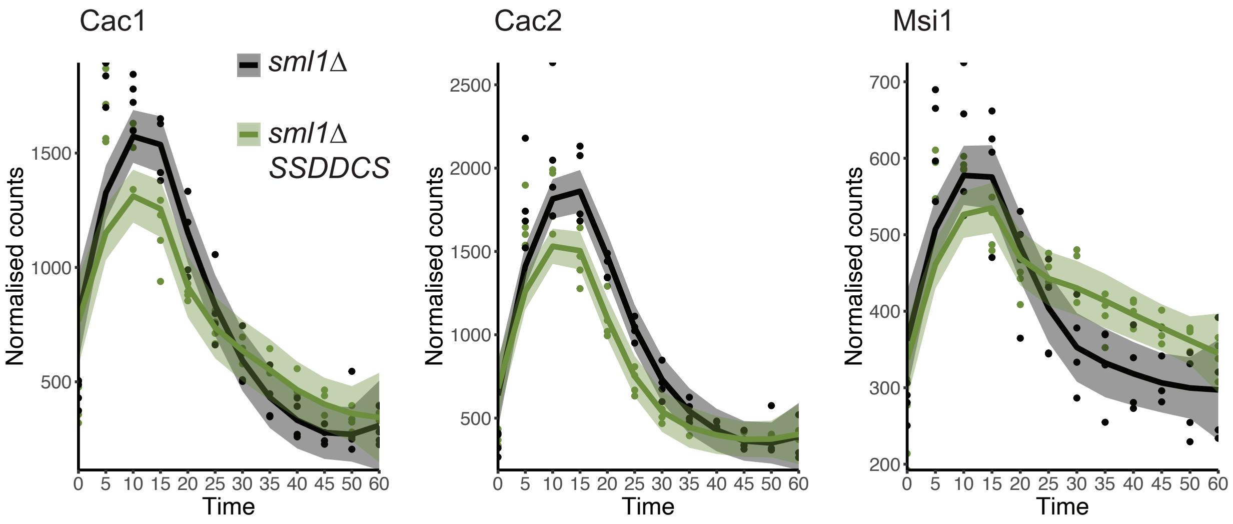

Supplementary Figure Legends

Fig. S1. Overexpression of SSDDCS does not affect the G1-S transition.

A. Budding index of the experiment from Figure 1A and 2A. Budding is an indicator of G1-CDK activity.
B. Gene expression (from the RNA-seq analysis, Figure 2) of the G1 cyclins (Cln1-3) and S-phase cyclins (Clb5-6) is the same between strains indicating that the G1-S transition is not affected in the *SSDDCS* strain.

Fig. S2. PCA analysis of batch effects in the RNA-seq datasets.

Principal component analysis (PCA) plot illustrating the clustering of individual replicates per time-point and strain. Samples are mostly clustered by time-point, illustrating the cell cycle nature of gene expression regulation in budding yeast n = 4.

Fig. S3. Analysis of the proximity of differentially expressed genes (DE) to origins, telomeres and centromeres.

A) Distribution of ΔT_{rep} (*sml1Δ* - *sml1Δ SSDDCS*) for all genes, divided into the k-means clusters from Figure 2C. The dotted line marks 0 (no difference in T_{rep}).

B) Distribution of gene distances to the closest origin for every gene in the genome, split by k-means cluster. NOT DE represents all non-differentially expressed genes. Dashed horizontal line marks the genome-wide median gene distance to the closest origin = 8.9 kb. p-values are from pairwise comparisons of each k-means cluster versus the non-DE genes using Wilcoxon rank sum test. **** $p < 0.0001$, ** $p < 0.01$.

C) Proportion of genes which are within or without sub-telomeric regions (less or more than 50kb away from the closest telomere, respectively). Vertical dashed line marks the percentage of all genes located in sub-telomeric regions = 12%. p-values are from an exact binomial test comparison to non-DE genes. **** $p < 0.0001$, ** $p < 0.01$.

D) Proportion of genes which are within or without sub-centromeric regions (less or more than 50kb away from the centromere, respectively). Vertical dashed line marks the percentage of all genes located in sub-centromeric regions = 14%. All groups had the expected proportion of genes in sub-centromeric regions.

Fig. S4. Analysis of the T_{rep} for all origins between the origin deletion strains.

Comparison of the T_{rep} for all origins between the *sml1Δ SSDDCS ΔARS816/818* strain (y-axis) and the *sml1Δ SSDDCS ΔARS1008/1009* strain (x-axis). This graph shows that the mutated origins deviate the most from the best fit line for all origins, indicating that the other origins have a similar T_{rep} when comparing between these strains.

Fig. S5. Overexpression of SSDDCS does not affect the expression of the CAF-1 complex.

Gene expression (from the RNA-seq analysis, Figure 2) of the CAF-1 complex components Cac1, Cac2 and Msi1.
