# Supplementary material for: Global early replication disrupts gene expression and chromatin conformation in a single cell cycle
Source: Genome Biol. 2022 Oct 17;23:217. doi: 10.1186/s13059-022-02788-7 (PMC9575230; doi:10.1186/s13059-022-02788-7)
Supplement: Supplementary file 2 — Additional file 2. Review history. [file 13059_2022_2788_MOESM2_ESM.docx]

Review History

**First round of review**

**Reviewer 1**

**Were you able to assess all statistics in the manuscript, including the appropriateness of statistical tests used?**

Yes, and I have assessed the statistics in my report.

**Comments to author:**

In this manuscript, the authors take advantage of their prior observation that overexpression of 6 limiting replication factors in budding yeast considerably shortens S phase by compressing the replication timing program, interpreted to be due to the fact that origins are no longer competing for limiting factors. They also disable the S phase checkpoint to avoid checkpoint activation that occurs in these strains. They then find that this affects transcription of 27% of genes, enriched for cell cycle regulated genes. An elegant part of this manuscript is that, by manipulating origins genetically to return genes to their original replication time, the authors can abrogate the aberrant expression. This demonstrates a causal link between replication time in the cell cycle and transcription. They go on to show that transcription changes are associated with transient alterations in nucleosome positions and TF binding that return to normal after S phase. Overall I find the results to be of high general interest. However, they are preliminary in terms of what the causal link is. In particular, I don't agree that the temporal order of replication is underpinning this transient wave, but rather something else that coordinates replication of a gene with other events in the cell cycle, which would be very interesting to follow up. I don't think the authors need to have all the answers in one paper, but they should be careful about how they are interpreting these interesting results.

- From the data shown in Figure 1 and 2, I see a few minutes advance of entry into S, a slight global compression of S phase, but the temporal order of replication is affected only in certain regions of the genome (contrary to the abstract). Thus the same general earlier vs. later replication is occurring across most of the genome, so it is misleading to say that there is no longer competition for limiting factors or that there is global early replication or that there is a role for the temporal order of origin firing. There is an advance in entry into S phase with some changes in temporal order. This to me needs to be incorporated into the interpretation of these data - it is as if the replication clock is out of synch with other clocks in the cell cycle, so that we end up with these transient alterations in gene expression that are re-set. Evidence is strong that it has something to do with coordinating when a gene replicates relative to the general cell cycle clock, but the evidence does not support a role for the temporal order of replication.

- To test our different interpretations (the authors' and mine), if the authors scale the relative times of replication, and then look at changes in the temporal order of replication, how does that affect their categories? If it is truly a role of the temporal order, then we should see some stratification by temporal order that differs from simply the advance in time from alpha factor release.

**Reviewer 2**

**Were you able to assess all statistics in the manuscript, including the appropriateness of statistical tests used?**

Yes, and I have assessed the statistics in my report.

**Comments to author:**

Replication of DNA in eukaryotes typically occurs with distinct timing where certain regions are replicated at different times during S phase. The reasons why replication follows a temporal program remains unclear, but a likely model - previously by the Zegerman lab - suggests that only a limited amount of DNA replication can be supported at any given time so as to not exhaust cellular resources, such as dNTPs. This current paper extends this work and shows that when origins are forced to fire early, there are significant alterations in gene expression. Changes in gene expression include inappropriate transcription of otherwise silenced genes. The authors show that misexpression is correlated with changes in chromatin and nucleosome positioning. Deletion of replication origins near to a misexpressed gene rescues the defect, indicating that timing of replication is likely causal. Finally, the authors compare their gene transcription data with published datasets or various deletion strains. They provide good evidence that mutations in the histone chaperone Caf1 phenocopy transcription defects they see when origins are forced to fire early. This leads the authors to a simple - yet satisfying - conclusion: transcription defects are caused by a chromatin assembly defect, when too many origins fire simultaneously.

Overall, I found this to be a very solid piece of work which should be of interest to a wide audience. The data is convincing, is well presented and provides important new insight into why replication timing may exist. I have a couple of suggestion that the authors may wish to consider:

First: does overexpression of histones and/or Caf1 suppress the transcription defect? Admittedly, this is a complex experiment given that mutant strain already has several genes overexpressed, and histone levels are controlled by Rad53, but such data would significantly strengthen the model.

Second: While SSDDCS overexpression leads to late origins firing early, it is less clear whether all origins fire simultaneously in a single cell. Can the authors discount that early replication of histone genes does not occur in a fraction of cells? Given that transcriptional defects may only occur in a sub-population of cells, this scenario may explain their results. Similarly, is expression of Caf1 downregulated by SSDDCS?

Third: Since the replication timing pattern is not altered in cac mutants, I find it puzzling that there is such good correlation between datasets. Can any insight be gained from in-depth comparison of affected genes in cac1 and SSDDCS mutants?

**Authors Response**

**Point-by-point responses to the reviewers’ comments:**

Reviewer #1:

In this manuscript, the authors take advantage of their prior observation that overexpression of 6 limiting replication factors in budding yeast considerably shortens S phase by compressing the replication timing program, interpreted to be due to the fact that origins are no longer competing for limiting factors. They also disable the S phase checkpoint to avoid checkpoint activation that occurs in these strains. They then find that this affects transcription of 27% of genes, enriched for cell cycle regulated genes. An elegant part of this manuscript is that, by manipulating origins genetically to return genes to their original replication time, the authors can abrogate the aberrant expression. This demonstrates a causal link between replication time in the cell cycle and transcription. They go on to show that transcription changes are associated with transient alterations in nucleosome positions and TF binding that return to normal after S phase. Overall I find the results to be of high general interest. However, they are preliminary in terms of what the causal link is. In particular, I don't agree that the temporal order of replication is underpinning this transient wave, but rather something else that coordinates replication of a gene with other events in the cell cycle, which would be very interesting to follow up. I don't think the authors need to have all the answers in one paper, but they should be careful about how they are interpreting these interesting results.

- From the data shown in Figure 1 and 2, I see a few minutes advance of entry into S, a slight global compression of S phase, but the temporal order of replication is affected only in certain regions of the genome (contrary to the abstract). Thus the same general earlier vs. later replication is occurring across most of the genome, so it is misleading to say that there is no longer competition for limiting factors or that there is global early replication or that there is a role for the temporal order of origin firing. There is an advance in entry into S phase with some changes in temporal order. This to me needs to be incorporated into the interpretation of these data - it is as if the replication clock is out of synch with other clocks in the cell cycle, so that we end up with these transient alterations in gene expression that are re-set. Evidence is strong that it has something to do with coordinating when a gene replicates relative to the general cell cycle clock, but the evidence does not support a role for the temporal order of replication.

*Reply: I think that this reviewer’s interpretation of the data is very similar to ours and we agree with many of the points being made here. I apologise if we were too brief in our description of the replication changes that occur in our experiments and we have amended the wording of the manuscript accordingly, including in the abstract. The key points we make clearer are as follows:*

*• The temporal order of origin firing has two outcomes: i) some origins fire earlier than others and ii) origin firing events are spread out over S-phase. In our SSDDCS strain we are affecting both of these outcomes. In Figure 1A, 9 out of 14 origins on Chr8 fire at the same time in early S-phase in the SSDDCS strain and when analysing all origins in Figure 1B, the first three quintiles of origins fire at the same, earliest timepoint, on average. Therefore, both figures show that at least 60% of origins fire at the same time in early S-phase in the SSDDCS strain. It is also the case that more origins fire in the SSDDCS strain, such as dormant origins (see ARS810 and ARS821 in Figure 1A). It is true that these origins aren’t quite as early as the other origins, but they fire early enough to be considered ‘early’ in the wild type strain, for example ARS821 fires in the SSDDCS strain at the same time as the early origin ARS815 in the control strain.*

*• The SSDDCS strain advances the replication time of all origins (except the ones that are already very early, Figure 1C), but of course non-origin regions by definition are replicated after the origins. When we analyse the whole genome in 1kb bins (Figure 1D), the vast majority of the genome including non-origin DNA is earlier replicating in the SSDDCS strain compared to the control strain.*

*• The temporal changes in genome duplication that are induced by the SSDDCS strain are quite profound and we now include a new Figure 1C to make this clearer. This figure shows that the earliest replicating regions of the genome (centromeres) remain early, while the normally latest replicating regions of the yeast genome, the telomeres, on average replicate as early as centromeres in the SSDDCS strain. So, together with Figure 1D, we consider it reasonable to conclude that the SSDDCS expression induces dramatic global changes in replication timing.*

*• In the comment “From the data shown in Figure 1 and 2, I see a few minutes advance of entry into S” we are not sure whether the reviewer is suggesting that in the SSDDCS strain we are inadvertently inducing an early S-phase by interfering with the G1-S transition. This is definitely not the case, which we now make clearer by including the budding index and the expression of the G1 and S-phase cyclins for the experiment in Figure 2 in Supplementary Figure 1.*

*As suggested by the reviewer, we have incorporated these points in more detail into the interpretation of the data – see in particular lines 121-140.*

- To test our different interpretations (the authors' and mine), if the authors scale the relative times of replication, and then look at changes in the temporal order of replication, how does that affect their categories? If it is truly a role of the temporal order, then we should see some stratification by temporal order that differs from simply the advance in time from alpha factor release.

*Reply: We are not making the case that the order in which parts of the genome is replicated is determining the gene expression changes. We make the case that the early replication of the genome affects gene expression, for example in Figure 4 when we make IME2 and NDT80 late replicating we return their expression back to normal.*

*If we separate the differentially expressed gene clusters by tREP as suggested, (see below) we see that, all genes, both differentially expressed (DE) and non-DE, replicate earlier in the SSDDCS strain. This is expected because the majority of the genome replicates earlier in the SSDDCS strain. Cluster 4 genes are on average earlier replicated than non-DE genes in the SSDDCS strain but not in the control strain. In addition, Cluster 1 and 5 genes are on average even earlier replicated than non-DE genes in the SSDDCS strain compared to control, but the other clusters are similar. As a result, I don’t think this corroborates or disagrees with the reviewer’s point as some DE gene clusters do change their relative replication timing compared to non-DE, while others don’t.*

*The simplest explanation of our data, in light of the striking correlation of our gene expression changes with histone chaperone mutants (Figure 5) and the global, transient disruption of nucleosome and TF positioning (Figure 5/6), is that global early replication doesn’t allow enough time for histone expression/assembly, leading to a transient window in S-phase when TF binding and transcription is perturbed (Figure 6F).*

Reviewer #2:

Replication of DNA in eukaryotes typically occurs with distinct timing where certain regions are replicated at different times during S phase. The reasons why replication follows a temporal program remains unclear, but a likely model - previously by the Zegerman lab - suggests that only a limited amount of DNA replication can be supported at any given time so as to not exhaust cellular resources, such as dNTPs. This current paper extends this work and shows that when origins are forced to fire early, there are significant alterations in gene expression. Changes in gene expression include inappropriate transcription of otherwise silenced genes. The authors show that misexpression is correlated with changes in chromatin and nucleosome positioning. Deletion of replication origins near to a misexpressed gene rescues the defect, indicating that timing of replication is likely causal. Finally, the authors compare their gene transcription data with published datasets or various deletion strains. They provide good evidence that mutations in the histone chaperone Caf1 phenocopy transcription defects they see when origins are forced to fire early. This leads the authors to a simple - yet satisfying - conclusion: transcription defects are caused by a chromatin assembly defect, when too many origins fire simultaneously.

Overall, I found this to be a very solid piece of work which should be of interest to a wide audience. The data is convincing, is well presented and provides important new insight into why replication timing may exist. I have a couple of suggestion that the authors may wish to consider:

First: does overexpression of histones and/or Caf1 suppress the transcription defect? Admittedly, this is a complex experiment given that mutant strain already has several genes overexpressed, and histone levels are controlled by Rad53, but such data would significantly strengthen the model.

*Reply: We agree this is an interesting experiment and we have spent some time working out whether it would be feasible. We are concerned that conditional overexpression of all four histones would be too toxic, as recent reports show that this creates major genome instability and difficulties in exiting G1 phase in a single cell cycle (Maya-Miles et al 2018 eLife). This would prevent us from conducting the S-phase expression analysis as described in this study. We are also not sure that over-expressing CAF-1 would lead to more histone deposition in the SSDDCS strain, as we think it’s likely that the histone supply is limiting not CAF-1.*

Second: While SSDDCS overexpression leads to late origins firing early, it is less clear whether all origins fire simultaneously in a single cell. Can the authors discount that early replication of histone genes does not occur in a fraction of cells? Given that transcriptional defects may only occur in a sub-population of cells, this scenario may explain their results. Similarly, is expression of Caf1 downregulated by SSDDCS?

*Reply: Early replication of histones in the SSDDCS strain (even if only in a fraction of cells) would lead to more histone expression, which would be the opposite phenotype to what we observe in this strain.*

*We now include data that shows that CAF-1 expression is not affected in the SSDDCS strain (see new Supplementary Figure 5).*

Third: Since the replication timing pattern is not altered in cac mutants, I find it puzzling that there is such good correlation between datasets. Can any insight be gained from in-depth comparison of affected genes in cac1 and SSDDCS mutants?

*Reply: Our model proposes that enforced early replication of the majority of the genome leads to a histone supply problem (too much nascent DNA in a short space of time, Figure 6F). We think there is a good correlation with chromatin assembly mutants because these mutants also lead to a histone supply problem on DNA (albeit over a normal length of S-phase).*

*We have not found any common features of the affected genes in the SSDDCS and histone chaperone mutant list that might suggest a special type of regulation of this gene set.*

**Second round of review**

**Reviewer 1**

The authors have satisfactorily addressed my concerns, as they pointed out we basically agreed and it was a matter of correcting the misleading wording, particularly in the abstract.

Reviewer 2

The authors have adequately addressed my comments and questions.

**Authors Response**

**Point-by-point responses to the reviewers’ comments:**

Reviewer #1

The authors have satisfactorily addressed my concerns, as they pointed out we basically agreed and it was a matter of correcting the misleading wording, particularly in the abstract.

Reviewer #2

The authors have adequately addressed my comments and questions.

*Reply: We kindly thank the reviewers for their comments.*
